# Effect of Genetic Variants on Rosuvastatin Pharmacokinetics in Healthy Volunteers: Involvement of *ABCG2*, *SLCO1B1* and *NAT2*

**DOI:** 10.3390/ijms26010260

**Published:** 2024-12-30

**Authors:** Eva González-Iglesias, Clara Méndez-Ponce, Dolores Ochoa, Manuel Román, Gina Mejía-Abril, Samuel Martín-Vilchez, Alejandro de Miguel, Antía Gómez-Fernández, Andrea Rodríguez-Lopez, Paula Soria-Chacartegui, Francisco Abad-Santos, Jesús Novalbos

**Affiliations:** 1 Clinical Pharmacology Department, Hospital Universitario de La Princesa, Instituto de Investigación Sanitaria La Princesa (IIS-Princesa), 28006 Madrid, Spain; egiglesias@salud.madrid.org (E.G.-I.);; 2 Pharmacology Department, Faculty of Medicine, Universidad Autónoma de Madrid (UAM), 28029 Madrid, Spain; 3 Centro de Investigación Biomédica en Red de Enfermedades Hepáticas y Digestivas (CIBERehd), Instituto de Salud Carlos III, 28029 Madrid, Spain

**Keywords:** pharmacogenetics, pharmacokinetics, statins, rosuvastatin, clinical trials, musculoskeletal side effects

## Abstract

Statins are the primary drugs used to prevent cardiovascular disease by inhibiting the HMG-CoA reductase, an enzyme crucial for the synthesis of LDL cholesterol in the liver. A significant number of patients experience adverse drug reactions (ADRs), particularly musculoskeletal problems, which can affect adherence to treatment. Recent clinical guidelines, such as those from the Clinical Pharmacogenetics Implementation Consortium (CPIC) in 2022, recommend adjusting rosuvastatin doses based on genetic variations in the *ABCG2* and *SLCO1B1* genes to minimize ADRs and improve treatment efficacy. Despite these adjustments, some patients still experience ADRs. So, we performed a candidate gene study to better understand the pharmacogenetics of rosuvastatin. This study included 119 healthy volunteers who participated in three bioequivalence trials of rosuvastatin alone or in combination with ezetimibe at the Clinical Trials Unit of the Hospital Universitario de La Princesa (UECHUP). Participants were genotyped using a custom OpenArray from ThermoFisher that assessed 124 variants in 38 genes associated with drug metabolism and transport. No significant differences were observed according to sex or biogeographic origin. A significant increase in t_1/2_ (*p_multivariate_*(*p_mv_*) = 0.013) was observed in the rosuvastatin plus ezetimibe trial compared with the rosuvastatin alone trials. Genetic analysis showed that decreased (DF) and poor function (PF) volunteers for the ABCG2 transporter had higher AUC_∞_/DW (adjusted dose/weight), AUC_72h_/DW and C_max_/DW compared to normal function (NF) volunteers (*p_mv_* < 0.001). DF and PF volunteers for SLCO1B1 showed an increase in AUC_72h_/DW (*p_mv_* = 0.020) compared to increased (IF) and NF individuals. Results for ABCG2 and SLCO1B1 were consistent with the existing literature. In addition, AUC_∞_/DW, AUC_72h_/DW and C_max_/DW were increased in intermediate (IA) and poor (PA) NAT2 acetylators (*p_mv_* = 0.001, *p_mv_* < 0.001, *p_mv_* < 0.001, respectively) compared to rapid acetylators (RA), which could be associated through a secondary pathway that was previously unknown.

## 1. Introduction

Cardiovascular disease (CVD) is a major global public health burden [1]. The main cause of CVD is high blood cholesterol, especially low-density lipoprotein (LDL), which is responsible for the formation of atherosclerotic plaques that can lead to heart attack or stroke [2]. Other risk factors for CVD include high blood pressure, smoking, sedentary lifestyle, obesity and unhealthy diet. Lifestyle changes are therefore essential for the prevention and treatment of CVD [3].

In many cases, these lifestyle changes need to be combined with pharmacological treatments to achieve target LDL levels. In their 2019 guidelines, the European Society of Cardiology and the European Atherosclerosis Society (ESC/EAS) recommended the use of statins as first-line therapy for the primary and secondary prevention of CVD since they improve lipid profiles and reduce cardiovascular morbidity and mortality [4]. Their mechanism of action is based on the inhibition of the key enzyme in the production of cholesterol in the liver, 3-hydroxy-3-methylglutaryl coenzyme A (HMG-CoA) reductase [5]. In 2012, around 10% of the Spanish population was receiving a statin for this indication [6]. There are currently seven statins on market—atorvastatin, simvastatin, pravastatin, pitavastatin, fluvastatin, lovastatin and rosuvastatin—all of which have different recommendations for use depending on cardiovascular risk [7].

This work will focus on rosuvastatin, which reaches maximum plasma concentration (C_max_) approximately 5 h after administration (t_max_), and it has an oral bioavailability of 20%. Its elimination half-life (t_1/2_) is between 18 and 20 h after a single dose [8,9]. Rosuvastatin is absorbed from the gastrointestinal tract after oral administration. Absorption is greatest on an empty stomach as it may be affected by the presence of food in the stomach [10]. Once absorbed, rosuvastatin binds to proteins in the blood and is transported to the liver via the OATP1B1 transporter (encoded by the *SLCO1B1* gene) and less commonly via SLC10A1, SLCO1B3 and SLCO2B1 [11]. The entry of rosuvastatin into the liver cells is essential since this is where its effect on cholesterol synthesis takes place.

Rosuvastatin is minimally metabolized in the liver. It can be oxidized by CYP2C19 and CYP2C9 enzymes to N-desmethyl rosuvastatin, which is approximately 50% less active than rosuvastatin. It can also be metabolized by UGT1A3 and UGT1A1 enzymes to rosuvastatin lactone, a secondary metabolite that is considered clinically inactive [12]. Since only 10% of rosuvastatin is metabolized, the remaining 90% is eliminated by biliary excretion via the BCRP transporter (encoded by the *ABCG2* gene), ABCB11 and ABCC2. These transporters are located on the hepatocyte membrane and extrude the drugs into the bile canaliculi. Inactive metabolites and a small amount of unchanged drug are excreted mainly in the bile and feces, although a small amount is excreted via the kidneys [12].

Despite the efficacy of statins, a significant percentage of patients experience significant adverse drug reactions (ADRs) that may limit adherence to treatment. Some ADRs are common (1–10%), such as headache, nausea or musculoskeletal disorders such as myalgia or arthralgia, but there are also others which, although less frequent, can be serious, such as myopathy, myositis, rhabdomyolysis or tendinopathy, sometimes complicated with rupture [8]. These statin-associated musculoskeletal side effects (SAMS) can affect adherence to treatment and ultimately compromise long-term efficacy [13].

In addition to statins, there are other drug treatments for cholesterol management, such as PCSK9 inhibitors or cholesterol absorption inhibitors (e.g., ezetimibe). These may be used in patients who cannot tolerate statins or in combination with statins in other patients who do not respond to statins alone and whose dose cannot be increased because of the risk of adverse effects [14,15,16].

Pharmacogenetic guidelines are being established to provide genotype-based dosing recommendations with the aim of preventing these ADRs [17,18]. The Clinical Pharmacogenetics Implementation Consortium (CPIC) in its statins guideline of 2022 includes recommendations for dose adjustment according to the pharmacogenetic phenotype of SLCO1B1 (for atorvastatin, simvastatin, rosuvastatin, fluvastatin, pitavastatin, pravastatin, lovastatin), ABCG2 (for rosuvastatin) and CYP2C9 (for fluvastatin) to prevent SAMS [13].

Although it is known that rosuvastatin treatment should be adjusted according to the pharmacogenetic phenotype of SLCO1B1 and ABCG2, many patients still experience complications with therapy. Therefore, the main objective of this study was to perform a candidate gene study to identify genes that may be associated with altered rosuvastatin kinetics. For this, we analyzed 124 variants in 38 genes in 119 healthy volunteers participating in three bioequivalence clinical trials of rosuvastatin alone or in combination with ezetimibe. This work is part of the Hospital Universitario de La Princesa’s Multidisciplinary Initiative for the Implementation of Pharmacogenetics (PriME-PGx) [19].

## 2. Results

### 2.1. Demographic Characteristics

A total of 119 healthy volunteers participated in the study, with similar distribution of sex (52% men and 48% women) and a median age of 26 years for men and 24 years for women (Table 1). Men and women showed significant differences in weight (*p *< 0.001), height (*p *< 0.001) and BMI (*p* = 0.008), with men being taller, heavier and having higher BMIs compared to women.

The population consisted of 69 (58%) Latin Americans and 50 (42%) Europeans. Europeans were significantly younger (*p *< 0.001), taller (*p *< 0.001) and with lower BMIs (*p *< 0.001) than Latin Americans (Table 1).

The two rosuvastatin-only studies included a total of 72 volunteers, while the rosuvastatin/ezetimibe study included 47 volunteers. Volunteers who participated in rosuvastatin/ezetimibe trial were older (*p *< 0.001) and had a higher BMI (*p *< 0.001) than those who received rosuvastatin alone (Table 1).

### 2.2. Pharmacokinetics

AUC_∞_ was 158.40 ± 74.41 h*ng/mL in men vs. 169.40 ± 53.43 h*ng/mL in women, AUC_72h_ was 153.43 ± 73.27 h*ng/mL in men vs. 159.50 ± 49.63 h*ng/mL in women, and C_max_ was 14.63 ± 7.04 ng/mL in men vs. 16.12 ± 5.65 ng/mL in women. No significant differences were observed.

After adjusting for dose/weight (DW), no differences were observed in the pharmacokinetic data according to sex (Table 2). Regarding biogeographical origins, Latin Americans presented longer t_1/2_ than Europeans (*p* = 0.008). T_max_ was similar in both groups, although with a significant increase (*p* = 0.036) in Latin Americans in comparison to Europeans. None of these results were held in the multivariate analysis.

Participants receiving rosuvastatin alone had higher mean AUC_72h_/DW values than those receiving rosuvastatin/ezetimibe (*p* = 0.029). Additionally, t_1/2_ was higher in rosuvastatin/ezetimibe (*p* = 0.014; *p_mv_* = 0.011, β = 5.274, R^2^ = 0.047) than in rosuvastatin alone (Table 2).

### 2.3. Pharmacogenetics

A significant increase was observed in AUC_∞_/DW (803.58 ± 256.17 h*ng*kg/mL*mg, *p *< 0.001; *p_mv_* < 0.001, β = 0.467, R^2^ = 0.264), AUC_72h_/DW (766.54 ± 289.52 h*ng*kg/mL*mg, *p *< 0.001; *p_mv_* < 0.001, β = 0.449, R^2^ = 0.302) and C_max_/DW (76.98 ± 31.87 ng*kg/mL*mg, *p *< 0.001; *p_mv_* < 0.001, β = 0.480, R^2^ = 0.292) in decreased function (DF) + poor function (PF) volunteers for ABCG2 transporter in comparison to normal function (NF) volunteers (Table 3).

A significant increase in AUC_72h_/DW was observed in SLCO1B1 DF + PF volunteers in comparison to increased function (IF) + NF (596.00 ± 221.31 h*ng*kg/mL*mg vs. 504.83 ± 216.89 h*ng*kg/mL*mg, *p* = 0.040, *p_mv_* = 0.020, β = 0.187, R^2^ = 0.302). Additionally, a clear trend of increased AUC_∞_/DW (613.46 ± 220.42 h*ng*kg/mL*mg, *p* = 0.060) and C_max_/DW (57.69 ± 22.19 ng*kg/mL*mg, *p* = 0.067) was also observed for SLCO1B1 DFs + PFs in comparison to IFs + NFs (530.36 ± 223.98 h*ng*kg/mL*mg for AUC_∞_/DW; and 49.59 ± 22.09 ng*kg/mL*mg for C_max_/DW) (Table 3).

A significant increase in AUC_∞_/DW (*p* = 0.004; *p_mv_* = 0.001, β = 0.398, R^2^ = 0.264), AUC_72h_/DW (*p *< 0.001; *p_mv_* = 0.001, β = 0.427, R^2^ = 0.302) and C_max_/DW (*p *< 0.001; *p_mv_* < 0.001, β = 0.507, R^2^ = 0.292) was observed in subjects who were intermediate acetylators (IA) for NAT2, and these parameters were even higher in those who were poor acetylators (PA) compared to rapid acetylators (RA) (Table 3).

For TPMT enzyme, a significant decrease in AUC_∞_/DW (*p* = 0.031), AUC_72h_/DW (*p* = 0.029, *p_mv_* = 0.049, β = −0.267, R^2^ = 0.302) and C_max_/DW (*p* = 0.004; *p_mv_* = 0.011, β = −0.382, R^2^ = 0.292) parameters was observed when subjects were intermediate metabolizers (IM) in comparison to normal metabolizers (NM) (Table 3).

*UGT2B15* rs1902023 T/G subjects showed a significant decrease in AUC_∞_/DW in comparison to T/T (*p* = 0.037, *p_mv_* = 0.046, β = −0.178, R^2^ = 0.264). *UGT2B15* rs1902023 also showed differences in AUC_72h_/DW values overall (*p* = 0.036), but significance was lost in pairwise comparison (Table 3).

T_max_ showed a significant decrease in the T/C genotype for *CES1* rs8192936 (*p* = 0.012, *p_mv_* = 0.019, β = −0.795, R^2^ = 0.068) in comparison to T/T, but it is not clinically relevant. A significant decrease in t_1/2_ was observed in C/T volunteers for *COMT* rs13306278 (*p* = 0.044) compared to C/C, but it is not maintained in multivariate analysis (Table 3).

T_max_ was increased in A/- subjects for *CYP3A43* rs61469810 in comparison to A/A (*p* = 0.017) and in *CYP4F2* rs2108622 G/G (*p* = 0.015) and G/A (*p* = 0.012) in comparison to A/A. *CYP4F2* rs3093105 showed differences in C_max_/DW values overall (*p* = 0.041), but significance was lost in pairwise comparison (Table 3).

A significant decrease in t_1/2_ is observed in *G6PD* rs1050828 G/A + A/A subjects (10.96 h (9.99–11.93), *p* = 0.039) in comparison to G/G and in *G6PD* rs1050829 A/G + G/G (11.08 h (10.29–12.66), *p* = 0.021) in comparison to A/A (Table 3). These differences were not maintained in the multivariate analysis.

Volunteers with a *UGT1A1* rs887829 C/T genotype showed a significant increase in T_max_ compared to C/C (*p* = 0.030, *p_mv_* = 0.037, β = 0.479, R^2^ = 0.068) and in *UGT1A34* rs2008584 A/G in comparison to A/A (*p* = 0.033). For *UGT1A6* rs10445704 A/A volunteers, a significant decrease in t_1/2_ (*p* = 0.036) in comparison to G/G was observed, and the A/G volunteers showed an increase in T_max_ (*p* = 0.045) in comparison to G/G (Table 3).

To summarize, only *ABCG2, SLCO1B1, NAT2, TPMT* and *UGT2B15* genes were significant for AUC and C_max_ in the multivariate analysis.

No significant differences were found when we analyzed the remaining genes involved in the metabolism or transport of rosuvastatin, but a trend toward an increase in AUC and C_max_ was observed in CYP2C9 poor metabolizers (PM) (Table 4). The results for the other pharmacogenes studied are shown in Supplementary Table S2.

### 2.4. Adverse Drug Reactions

ADRs were present in 15 volunteers (12.60%) in total (six in clinical trials A and C, and three in clinical trial B, *p* = 0.613), 12 of whom suffered one ADR, two of whom suffered two ADRs, and only one suffered three ADRs. Headache was the most common ADR (52.64%), followed by dizziness (15.80%), fatigue (10.52%), muscular fatigue, constipation, liquid stools and exanthema due to possible allergic drug reaction (5.26%). Women had a non-significantly higher incidence of ADRs than men (66.67% women vs. 33.33% men, *p* = 0.167). European volunteers had a non-significantly higher incidence of ADRs in comparison to Latin American volunteers (66.67% European vs. 33.33% Latin American, *p* = 0.581). Volunteers in the two rosuvastatin-alone trials had nine ADRs, and those in the combined trial had six (*p* = 1.00).

There were no significant differences in pharmacokinetic parameters according to ADR incidence. When comparing ADRs with the genotype or phenotype of the main outcomes obtained (*ABCG2*, *SLCO1B1*, *NAT2, TPMT* and *UGT2B15* genes), no significant differences were found in the chi-squared tests.

## 3. Discussion

Statins are widely prescribed for the treatment of hypercholesterolemia and have demonstrated overwhelming benefits in reducing cardiovascular morbidity and mortality. However, a significant percentage of patients discontinue treatment due to the development of adverse events. In recent years, the goal has been to implement personalized medicine that allows treatment tailoring to improve adherence and reduce ADRs.

Regarding statins, CPIC published its guideline on simvastatin in 2012 [20], which was updated in 2014 [21]. In 2022, a guideline including the seven statins was finally published. In the case of rosuvastatin, CPIC recommends dose adjustment based on the pharmacogenetic phenotype of the transporters encoded by the *ABCG2* and *SLCO1B1* genes [13]. The aim of this work was to demonstrate the effect of these and other pharmacogenes on the kinetics of rosuvastatin, as well as other factors such as sex, biogeographical origin or co-administration with ezetimibe.

Similar to previous studies, men exhibited higher weight, height and BMI than women [22,23]. Although no differences in pharmacokinetics were observed based on sex when the parameters were not dose/weight adjusted, it was finally decided to adjust them due to the weight differences. With this dose/weight adjustment, we were able to standardize the results and avoid possible interferences to make accurate and fair comparisons between different individuals and groups. With these parameters, no differences were observed based on sex, which is consistent with the summary of product characteristics and the literature [8,24]. T_max_ results are consistent with the AEMPS summary of product characteristics; however, the t_1/2_ reflected is 19 h, which is slightly higher than that shown in this article [8].

In our research, differences in rosuvastatin pharmacokinetics between biogeographical origins were found in univariate analysis, with Latin Americans having higher T_max_ and t_1/2_ than Europeans. However, this was not confirmed in the multivariate analysis, suggesting that these differences are due to other factors. No association between differences in rosuvastatin pharmacokinetics and European or Latin American origin has been described, but the interaction with Asians and Africans has been extensively studied [25,26,27,28]. Additionally, the frequency of phenotypes for the principal genes and transporters involved in statin metabolism differs between European and Latino populations, which could perhaps explain the small differences we observed between origins and why these results are not maintained in the multivariate analysis [29].

The combination of rosuvastatin with another lipid-lowering drug, such as ezetimibe, has been extensively studied to improve the efficacy of treatment in patients who do not reach target cholesterol levels with statins [27]. In this study, a decrease in AUC_72h_/DW and an increase in t_1/2_ were found in the combined assay compared to rosuvastatin alone. This seems to indicate a lower exposure to the drug and a slower elimination in combination treatment. There are no results in the literature to support the results obtained [6,30,31,32,33], and, furthermore, these results contradict the drug label [8,9]. In this document, it is reported that with concomitant use of rosuvastatin and ezetimibe, the AUC of rosuvastatin resulted in a 1.2-fold increase in AUC with respect to the administration of rosuvastatin alone in hypercholesterolemic subjects [34]. Given these results, it is possible that the observed differences may be influenced by the individual characteristics of the subjects who participated in the different trials rather than the specific study design itself [32]. For example, one explanation for this could be that the combined study included more Latin Americans, who also had a higher t_1/2_. Furthermore, it is pertinent to note that a significant disparity in the analytical determination between the two types of studies exists; the study examining rosuvastatin alone was conducted in 2013, whereas the combined study was conducted in 2022. This temporal difference could be associated with changes in analytical methods, equipment or other procedural factors, despite their apparent similarity on paper.

For the *ABCG2* gene, a 60% increase in AUC_∞_/DW, AUC_72h_/DW and C_max_/DW was observed in DF and PF individuals compared to NFs. These results indicate that individuals with reduced ABCG2 transporter function have greater drug accumulation, as reflected by an increase in both AUC and C_max_. These findings are consistent with previous studies as reported in several investigations [13,35,36,37,38] and the summary of product characteristics [8]. This information is what has motivated the CPIC to state that the ABCG2 pharmacogenetic phenotype should be considered when adjusting the dose of rosuvastatin. An initial dose of no more than 20 mg is recommended in individuals with DF or PF, and the use of therapeutic alternatives or combinations is considered if a higher dose is required to achieve the desired efficacy [13].

The *SLCO1B1* gene, which encodes the OATP1B1 transporter protein, plays an important role in the pharmacokinetics of several statins, including rosuvastatin. This protein plays a crucial role in transporting rosuvastatin to the liver, where it exerts its lipid-lowering effect. Genetic variants in *SLCO1B1* have been associated with changes in rosuvastatin plasma concentrations [39,40], which eventually led to it being one of the genes, along with *ABCG2*, considered by CPIC for dose adjustment [13]. In the results obtained, a significant 20% increase in AUC_72h_/DW and a tendency in AUC_∞_/DW and C_max_/DW was observed in DF + PF volunteers in comparison to IF + NF. Phenotypes were grouped in this way since they share the same recommendations. One factor that may explain why these differences were not significant for all the parameters is the small number of subjects with poor function. In addition, articles found in the literature indicate that changes in the function of this transporter do not have a significant effect on the kinetics of rosuvastatin [27,41]. This may suggest that the influence of this transporter on rosuvastatin does not translate into such relevant differences in the efficacy or safety of the drug, and therefore the influence is less important than with other statins, such as simvastatin or atorvastatin, where the relationship with kinetics and ADRs seem to be clearer [27,39]. The literature indicates the importance of this gene in the risk of myopathy in rosuvastatin treatment when this enzyme loses its function [38,40]. This may be the reason why CPIC includes this gene for rosuvastatin dose adjustment, although there are few studies showing a clear increase in AUC and Cmax in subjects carrying non-functional polymorphisms [39,40]. In conclusion, although dose adjustment based on the *SLCO1B1* and *ABCG2* gene is a recommended practice, the effect of *SLCO1B1* on rosuvastatin could be small compared to other statins.

NAT2 (N-acetyltransferase 2) is a key enzyme in the metabolism of several xenobiotics, including drugs. This enzyme is involved in acetylation, a phase II metabolic process that facilitates the elimination of compounds from the body [42]. Genetic variations in the *NAT2* gene determine the acetylation capacity of each individual, dividing them into three phenotypes: rapid, intermediate and poor acetylators. Rapid acetylators have a high capacity to metabolize drugs rapidly, which can reduce the efficacy of treatment as the drug is inactivated before it can exert its full therapeutic effect. On the other hand, intermediate and poor acetylators have a lower capacity for acetylation, leading to the accumulation of drug metabolites, and therefore they present a higher risk of toxicity [42]. Our results showed an increase of 30% in AUC_∞_/DW, AUC_72h_/DW and C_max_/DW in individuals with IA phenotype and a 50% increase in PAs. This has not been reported for rosuvastatin in previous studies but has been observed in previous articles with drugs such as isoniazid [43], rivaroxaban [44], diazepam [45] and rasagiline [46]. This finding suggests that variants in the *NAT2* gene may play a role in the pharmacokinetics of rosuvastatin or some intermediate metabolite. Unfortunately, after an exhaustive search in various databases, we have not found a clear explanation for the results obtained. So, further studies with higher number of subjects and functional studies are needed to better understand its role.

Thiopurine methyltransferase (TPMT) is an enzyme that has been associated with changes in plasma thiopurine levels [47]. Surprisingly, we found a decrease in AUC in intermediates. To date, no association has been described between the *TPMT* gene and the kinetics of rosuvastatin or any of the other statins on the market. Finally, UDP-glucuronosyltransferases (UGTs) are enzymes responsible for glucuronidation, an important process in the metabolism and elimination of many drugs. Overall, there is not much evidence linking UGTs to significant alterations in rosuvastatin kinetics. Furthermore, since the differences were observed in only one pharmacokinetic parameter for each gene of the family studied, it appears that UGTs do not play an important role in the metabolism of rosuvastatin [48].

## 4. Materials and Methods

### 4.1. Study Population

The participants of the present study were healthy volunteers who participated in three bioequivalence clinical trials of rosuvastatin (trials A and B) and rosuvastatin/ezetimibe (trial C) conducted at the Clinical Trials Unit of the Hospital Universitario de La Princesa (UECHUP), Madrid (Spain) during the years 2013 and 2022. The EU-CT numbers of the clinical trials were 2013-002047-28 (A), 2013-001078-15 (B) and 2022-501862-24-00 (C).

The main inclusion criteria were men or women aged 18–55 years, free of organic or psychological disorders; with normal vital signs, electrocardiogram and physical examination; and no significant abnormalities in hematology, coagulation, biochemistry, serology and urinalysis. Exclusion criteria were as follows: having received any medication within the two days prior to the start of the study, having a body mass index (BMI) outside the range of 18.5 to 30.0, being pregnant or breastfeeding, having a history of drug sensitivity, having a positive drug screen, smoking or alcoholism, having donated blood in the previous month, and having participated in another study with investigational drugs in the previous three months. Their weight and height were measured during the screening process. Healthy volunteers provided their biogeographical origin, biological sex and age.

All three studies were approved by the Spanish Agency of Medicines and Healthcare Products (AEMPS) and the Research Ethics Committee (CEIm) of the Hospital Universitario de La Princesa. The trials were conducted in accordance with Spanish legislation, the International Council for Harmonisation (ICH) guidelines on good clinical practice [49], the revised Declaration of Helsinki [50] and the Royal Decree on Biological Samples [51]. Volunteers were informed about this genetic study, and those who freely chose to participate signed a specific informed consent for the pharmacogenetic study (code SFC-FG-2020-1, IRB/Code: 4176), which was evaluated by the CEIm of the Hospital Universitario La Princesa and approved on 9 July 2020. Finally, 119 volunteers signed and participated in this pharmacogenetic study.

### 4.2. Study Design

Data were obtained from three bioequivalence clinical trials comparing a reference and test formulation of rosuvastatin and rosuvastatin/ezetimibe. These trials were phase I, open-label, single-center, crossover, randomized, two-sequence, two-period studies in which a single oral dose was administered on an empty stomach. In clinical trials A and B, a single dose of rosuvastatin 20 mg was administered, whereas in clinical trial C, a rosuvastatin/ezetimibe 20 mg/10 mg tablet was administered. At least 21 blood samples were extracted from each subject from pre-dose to 72 h after drug intake in each period for rosuvastatin and ezetimibe quantification.

### 4.3. Pharmacokinetic Analysis

Clinical laboratory analyses and determinations of plasma levels of rosuvastatin and ezetimibe were outsourced in the three studies. Drug determinations for pharmacokinetic analyses were performed after liquid–liquid extraction procedure using tert-butyl methyl ether by high-performance liquid chromatography coupled with tandem mass spectrometry (HPLC-MS/MS), a method validated according to the EMA guidelines [52].

WinNonLin Professional version 2.0 software (Scientific Consulting, Inc., Cary, NC, USA) for clinical trials A and B and 8.3 software for clinical trial C was used to calculate pharmacokinetic parameters according to a non-compartmental model. C_max_ and T_max_ were obtained directly from the plasma concentration–time curves. The area under the curve (AUC) between pre-dose and the last time of concentration measurement (t), 72 h (AUC_72h_), was calculated using the trapezoidal method. The remaining AUC from t to infinity (AUC_∞_) was calculated as the ratio C_t_/K_e_, where C_t_ is the last detectable concentration and K_e_ is the elimination slope, obtained by linear regression of the log-linear part of the concentration–time curve. In addition, t_1/2_ was calculated as ln2/K_e_. The test formulation was found to be bioequivalent to reference formulations, Lipocomb^®^ (trial C) and Crestor^®^ (trial A and B); however, to simplify the study, we only analyzed the data from the reference formulations.

### 4.4. Genotyping and Phenotyping

DNA was extracted from peripheral blood using a Maxwell^®^ RSC instrument (Promega, Madison, WI, USA). DNA concentration was measured using a Qubit 3.0 fluorometer (ThermoFisher, Waltham, MA, USA). Genotyping was performed on a QuantStudio 12k Flex real-time PCR system (Applied Biosystems, ThermoFisher, USA) using an OpenArray thermal block and a custom array that includes 124 variants in 38 genes related to drug metabolism or transport (Supplementary Table S1). Techniques were carried out in the pharmacogenetics unit of the Clinical Pharmacology Department of the Hospital Universitario de La Princesa.

Genotype-informed phenotypes for metabolizing enzymes or transporters were inferred according to PharmVar [53] and PharmGKB [54] core allele rules and according to the CPIC or the Dutch Pharmacogenetic Working Group (DPWG) guidelines for the following genes: *ABCG2* [13], *CYP2B6* [55], *CYP2C19* [55], *CYP2C9* [13], *CYP2D6* [55], *CYP3A5* [56], *SLCO1B1* [13] and *UGT1A1* [57]. Phenotypes were classified in ultrarapid, rapid, normal, intermediate and poor metabolizers (UM, RM, NM, IM, PM, respectively) for metabolizing enzymes. For transporters, the function was classified as increased, normal, decreased or poor function (IF, NM, DF, PF, respectively). *CYP2D6* deletion (*5), duplication and the presence of hybrid structures were analyzed using two TaqMan^®^ copy number variation assays targeting exon 9 and 5′ untranslated region (UTR) (Applied Biosystems, Foster City, CA, USA), as previously described [23]. The CYP2C8 phenotype was established as previously described by Campodonico et al. in 2022 [58]. *NAT2* alleles were defined according to the Arylamine N-acetyltransferase Gene Nomenclature Committee and were assigned as rapid acetylators (RA), intermediate acetylators (IA) and poor acetylators (PA) [59,60]. The remaining variants were analyzed individually.

### 4.5. Statistical Analysis

SPSS version 29.0 software (SPSS Inc., Chicago, IL, USA) was used for statistical analysis. AUC_∞,_ AUC_72h_ and C_max_ variables were adjusted for the dose/weight (DW) ratio to correct the effect of dose and weight on drug exposure. Normality of the data set was checked using the Shapiro–Wilk test. All pharmacokinetic parameters were log-transformed to normalize the distributions. Firstly, univariate analysis was performed for demographic characteristics (age, weight, height and BMI) and pharmacokinetic parameters by sex, biogeographic group and co-administration with ezetimibe. Additionally, pharmacokinetic parameters were also analyzed according to genotypes and phenotypes. T-tests were used to compare means for variables with two categories and ANOVA tests for variables with three or more categories, followed by a Bonferroni post hoc test. Where parametric tests were not applicable, the Mann–Whitney U test (for two categories within a variable) or a Kruskal–Wallis analysis of variance test (for three or more categories within a variable) was used. The *p*-value used for statistical significance was *p *< 0.05. Mean and standard deviation (SD) were reported for variables with normal distributions, and median and interquartile range (IQR, or the difference between the 1st and 3rd quartiles) were reported for those with non-normal distribution. Secondly, parameters with *p *< 0.05 in t-tests, ANOVA or non-parametric tests were entered as independent variables in a multivariate analysis performed by linear regression. To achieve this, those variables with more than two categories must first be dichotomized. Significant relationships (*p_multivariate (mv)_* <0.05) were indicated by the non-standardized β coefficient and R^2^ values. Thirdly, for safety evaluation, differences in ADR incidence were analyzed according to sex, biogeographic group and co-administration with ezetimibe, AUC_∞_, AUC_72h_ and C_max_ and also according to genotypes and phenotypes that were significant in the multivariate analysis of pharmacokinetic parameters (*ABCG2*, *SLCO1B1*, *NAT2*, *TPMT* and *UGT2B15* genes).

### 4.6. Limitations

The small number of subjects included in this study was a limitation due to the reduced statistical power. This is a single-dose study, which may not reflect steady-state pharmacokinetics achieved during long-term therapy. Since this was a study of healthy volunteers, the effect of variants on drug efficacy was not observed. Some of the genes that were analyzed, such as *ABCB1*, *ABCC2* or some *UGTs*, do not have well-characterized variant effects or defined alleles. In the case of the transporters, as they are expressed in different parts of the organism, it is more difficult to know whether it can affect pharmacokinetics. Finally, because this is a candidate gene study with selected variants for each gene, the information about other variants that may also alter the kinetics of rosuvastatin was lost.

### 4.7. Conclusions

Volunteers with DF or PF phenotypes for ABCG2 and SLCO1B1 have greater accumulation of rosuvastatin, although SLCO1B1 appears to have a minor clinical impact on rosuvastatin pharmacokinetics compared to other statins. An association between the *NAT2* gene and rosuvastatin kinetics is observed, possibly through an associated secondary pathway, currently unknown. The potential involvement of TPMT in the pharmacokinetics of statins is currently unknown. Further studies are required to validate these findings.

## Figures and Tables

**Table 1:** Demographic characteristics regarding sex, biogeographical origin and drug co-administration.

	N	Age (Years)	Weight (kg)	Height (m)	BMI (kg/m^2^)
Sex					
Men	62	26.00 (24.00–31.00)	**75.20 (66.95–80.63)**	**1.75 (0.66)**	**24.27 (2.69)**
Women	57	24.00 (22.00–31.00)	58.50 (55.40–64.75) *	1.62 (0.67) *	22.97 (2.58) *
Biogeographic origin					
European	69	**24.00 (22.00–28.50)**	64.70 (57.85–75.05)	**1.70 (8.76)**	**22.86 (2.55)**
Latin American	50	28.00 (24.75–35.25) *^1^	68.75 (59.55–78.40)	1.66 (9.43) *^1^	24.73 (2.56) *^1^
Drug co-administration					
Rosuvastatin	72	**24.00 (22.00–28.00)**	64.70 (57.78–74.15)	1.69 (8.90)	**22.85 (2.44)**
Rosuvastatin/ezetimibe	47	28.00 (25.00–37.00) *^2^	71.00 (59.90–79.90)	1.67 (9.66)	24.87 (2.66) *^2^

Data is shown as median (Q25–75) for non-normal distributions and mean (standard deviation) for normal distributions. BMI: body mass index. * *p* < 0.05 compared to men. *^1^ *p* < 0.05 compared to European. *^2^ *p* < 0.05 compared to rosuvastatin. Significant results are shown in bold.

**Table 2:** Rosuvastatin pharmacokinetics based on sex, biogeographical origin and drug co-administration.

	N	AUC_∞_/DW (h*ng*kg/mL*mg)	AUC_72h_/DW (h*ng*kg/mL*mg)	C_max_/DW (ng*kg/mL*mg)	t_1/2_ (h)	T_max_ (h)
Sex						
Men	62	580.98 (265.16)	562.46 (261.07)	53.71 (25.65)	14.59 (13.09–17.88)	5.50 (4.00–5.50)
Women	57	507.42 (161.27)	478.10 (151.38)	48.45 (17.56)	15.85 (12.38–22.51)	5.50 (5.50–5.50)
Biogeographic group						
European	69	563.59 (220.69)	545.10 (218.79)	51.92 (23.40)	**14.18 (12.07–18.18)**	**5.50 (3.75–5.50)**
Latin American	50	521.13 (227.76)	490.24 (217.11)	50.19 (20.65)	16.12 (13.89–20.89) *	5.50 (5.50–5.50) *
Drug co-administration						
Rosuvastatin	72	576.85 (233.71)	**556.95 (228.13)**	52.19 (22.99)	**14.38 (12.17–18.19)**	5.50 (4.63–5.50)
Rosuvastatin/ezetimiba	47	498.11 (200.65)	468.58 (194.21) *^1^	49.66 (21.13)	16.00 (13.87–22.33) *^1^	5.50 (5.00–5.50)

Data is shown as mean (standard deviation) for normal distribution and median (Q25–Q75) for non-normal distribution. AUC_∞_/DW: area under the plasma concentration–time curve from time zero to infinity, dose/weight corrected. AUC_72h_/DW: area under the plasma concentration–time curve from time zero to 72 h, dose/weight corrected. C_max_/DW: maximum plasma concentration, dose/weight corrected. t_1/2_: half-life. T_max_: time to reach maximum plasma concentration. * *p* < 0.05 compared to Europeans. *^1^ *p* < 0.05 compared to rosuvastatin. Significant results are shown in bold. Underlined: multivariate *p*-value (*p_mv_*) < 0.05.

**Table 3:** Rosuvastatin pharmacokinetic parameters with significant differences based on genotypes or phenotypes.

	N	AUC_∞_/DW (h*ng*kg/mL*mg)	AUC_72h_/DW (h*ng*kg/mL*mg)	C_max_/DW (ng*kg/mL*mg)	t_1/2_ (h)	T_max_ (h)
ABCG2		***p <* 0.001** ^@^	***p <* 0.001** ^@^	***p < * 0.001 ** ^@^	*p =* 0.154 ^@1^	*p =* 0.680 ^@^
NF	104	**508.56 (191.59)**	**486.79 (183.09)**	**47.47 (17.79)**	15.54 (12.92–19.77)	5.50 (4.50–5.50)
DF	14	790.77 (270.32) *	751.81 (294.56) *	75.51 (32.54) *	13.78 (11.76–15.85)	5.50 (5.50–5.50)
PF	1	982.88 *	972.79 *	97.61 *	13.10 ^#^	5.50 ^#^
SLCO1B1		*p =* 0.131 ^@1^	*p =* 0.099 ^@1^	*p =* 0.147 ^@1^	*p =* 0.242 ^@1^	*p =* 0.892 ^@1^
IF	2	414.54 (162.73)	406.52 (166.25)	38.76 (18.23)	11.67 (10.70–11.67) ^#^	5.50 ^~^
NF	91	532.90 (225.13)	506.99 (218.08)	49.83 (22.19)	15.66 (12.87–19.86)	5.50 (5.00–5.50)
DF	23	603.36 (219.62)	586.35 (221.07)	57.10 (22.50)	14.89 (13.56–18.19)	5.50 (5.50–5.50)
PF	1	845.8	817.76	71.19	18.17 ^#^	1.50 ^#^
NAT2		***p =*** **0.004**	***p*** **< 0.001**	***p*** **< 0.001**	*p =* 0.297	*p =* 0.825
RA	9	**377.72 (167.56)**	**327.32 (130.58)**	**30.59 (15.43)**	15.82 (13.24–30.05)	5.50 (4.25–5.50)
IA	44	515.16 (164.21) *^1^	491.61 (152.43) *^1^	49.21 (15.72) *^1^	15.98 (13.17–19.34)	5.50 (5.13–5.50)
PA	64	574.79 (230.97) *^1^	555.43 (230.66) *^1^	54.52 (24.88) *^1^	14.41 (12.03–18.66)	5.50 (4.63–5.50)
TPMT		***p =* 0.031**	***p =* 0.029**	***p = * 0.004**	*p =* 0.277	*p =* 0.832
NM	108	**556.50 (228.07)**	**532.68 (222.88)**	**52.34 (22.41)**	14.73 (12.70–18.19)	5.50 (5.00–5.50)
IM	7	401.58 (162.75) *^2^	383.00 (162.36) *^2^	33.20 (16.47) *^2^	16.30 (14.72–19.91)	5.50 (2.50–5.50)
*UGT2B15* rs1902023		***p =*** **0.018**	***p =*** **0.036** **^$^**	*p =* 0.059	*p =* 0.306	*p =* 0.715
T/T	21	**632.37 (257.68)**	**593.62 (265.45)**	55.94 (26.16)	15.66 (13.52–24.32)	5.50 (3.75–5.50)
T/G	64	500.81 (207.31) *^3^	**480.85 (200.94)**	47.62 (22.64)	14.53 (12.25–18.65)	5.50 (4.13–5.50)
G/G	34	**576.85 (216.78)**	**555.39 (209.27)**	54.97 (17.81)	15.06 (12.97–18.52)	5.50 (5.50–5.50)
*UGT1A1 rs887829*		*p =* 0.374	*p* = 0.493	*p =* 0.879	*p =* 0.224	***p =*** **0.013**
C/C	56	559.68 (218.68)	533.14 (217.37)	52.30 (24.17)	15.84 (13.03–19.81)	**5.50 (4.00–5.50)**
C/T	53	550.81 (242.42)	527.65 (234.70)	50.97 (21.46)	14.67 (12.86–20.05)	5.50 (5.50–5.50) *^4^
T/T	10	440.93 (104.36)	430.24 (103.38)	46.12 (14.24)	13.90 (11.63–16.12)	**5.50 (3.04–5.50)**
*CES1* rs8192936		*p =* 0.894	*p =* 0.818	*p =* 0.566	*p =* 0.311	***p =* 0.015**
T/T	16	526.44 (194.34)	493.66 (195.47)	46.81 (21.11)	15.28 (13.22–17.51)	**5.50 (5.50–5.50)**
T/C	54	548.81 (227.12)	522.09 (217.97)	51.66 (21.42)	15.98 (13.23–22.38)	5.50 (3.88–5.50) *^5^
C/C	49	548.68 (232.69)	531.27 (230.22)	52.10 (23.66)	14.45 (12.53–17.75)	**5.50 (5.25–5.50)**
*COMT rs13306278*		*p =* 0.832	*p =* 0.749	*p =* 0.852	***p =* 0.027**	*p =* 0.782
C/C	93	546.52 (228.80)	522.03 (225.71)	52.00 (23.21)	**15.90 (13.15–20.33)**	5.50 (4.75–5.50)
C/T	23	560.81 (200.03)	539.88 (187.79)	49.54 (18.29)	13.27 (11.98–15.66) *^6^	5.50 (3.50–5.50)
T/T	2	506.81 (293.23)	480.43 (270.78)	43.44 (24.85)	**12.55 (10.70–14.39) ^#^**	5.50 (5.50–5.50)
*CYP3A43* rs61469810		*p =* 0.278	*p =* 0.218	*p =* 0.070	*p =* 0.055	***p =*** **0.017**
A/A	102	548.87 (208.76)	527.12 (208.09)	52.53 (22.42)	14.56 (12.64–18.35)	**5.50 (4.50–5.50)**
A/-	17	527.04 (305.92)	491.65 (280.67)	43.17 (19.69)	16.38 (15.62–21.33)	5.50 (5.50–6.00) *^7^
*CYP4F2* rs2108622		*p =* 0.871	*p =* 0.843	*p =* 0.846	*p =* 0.686	***p =*** **0.035**
G/G	55	539.87 (204.64)	520.43 (197.03)	50.04 (18.05)	14.39 (12.87–17.73)	5.50 (5.50–5.50) *^8^
G/A	50	551.56 (249.69)	521.63 (246.94)	52.82 (26.51)	15.75 (12.92–21.26)	5.50 (5.50–5.50) *^8^
A/A	10	501.63 (182.16)	481.69 (177.61)	48.06 (22.03)	15.66 (13.47–20.87)	**4.00 (2.75–5.50)**
*CYP4F2* rs3093105		*p =* 0.074	*p =* 0.089	***p =* 0.041 ^$^**	*p =* 0.752	*p =* 0.449
T/T	78	527.08 (207.61)	503.98 (205.25)	**49.42 (21.02)**	14.84 (12.81–18.49)	5.50 (5.50–5.50)
T/G	33	616.16 (256.74)	588.44 (248.76)	**58.42 (24.67)**	15.66 (12.97–20.65)	5.50 (3.50–5.50)
G/G	7	460.11 (165.43)	445.88 (160.74)	**39.83 (15.44)**	14.72 (12.19–17.03)	5.50 (3.00–5.50)
*G6PD rs1050828*		*p =* 0.144 ^@2^	*p =* 0.120 ^@2^	*p =* 0.067 ^@2^	***p =* 0.039** ^@2^	*p =* 0.483 ^@2^
G/G	115	538.13 (217.53)	514.82 (212.26)	50.51 (21.42)	**15.23 (13.01–18.82)**	5.50 (5.00–5.50)
G/A	1	470.85	461.54	53.07	11.93 ^#^*^9^	3.00 ^#^
A/A	1	1223.73	1218.79	126.33	9.99 ^#^*^9^	5.50 ^#^
*G6PD rs1050829*		*p =* 0.315	*p =* 0.251	*p =* 0.150	***p* = 0.021**	*p =* 0.610
A/A	114	538.45 (217.11)	514.05 (211.14)	50.21 (21.27)	**15.40 (13.08–19.60)**	5.50 (5.00–5.50)
A/G	2	645.17 (246.52)	637.59 (248.96)	66.58 (19.11)	11.87 (11.80–11.93) ^#^*^10^	4.25 (3.00–5.50) ^#^
G/G	3	756.90 (423.72)	749.14 (426.64)	78.11 (44.64)	10.58 (9.99–13.39) ^#^*^10^	5.50 (3.17–5.50) ^#^
*UGT1A34* rs2008584		*p =* 0.525	*p =* 0.433	*p =* 0.474	*p =* 0.136	***p =*** **0.035**
A/A	37	579.37 (232.93)	556.05 (230.24)	54.36 (24.35)	15.23 (13.58–20.40)	**5.50 (3.50–5.50)**
A/G	58	525.19 (225.56)	497.73 (218.15)	48.18 (20.19)	15.84 (12.81–20.28)	5.50 (5.50–5.50) *^11^
G/G	23	545.62 (211.49)	530.01 (207.97)	53.51 (23.95)	13.87 (12.42–15.96)	**5.50 (3.50–5.50)**
*UGT1A6* rs10445704		*p =* 0.435	*p =* 0.517	*p =* 0.393	***p =*** **0.043**	***p =*** **0.037**
G/G	48	553.16 (230.47)	523.70 (228.64)	50.50 (23.56)	**15.77 (13.20–21.88)**	**5.50 (4.00–5.50)**
A/G	57	556.17 (226.23)	534.48 (218.91)	53.03 (21.78)	**15.42 (12.98–19.17)**	5.50 (5.50–5.50) *^12^
A/A	13	472.04 (196.68)	459.65 (193.98)	44.45 (19.41)	12.42 (10.85–15.60) *^12^	**5.50 (3.50–5.50)**

Data are shown as mean (standard deviation) for normal distribution and median (Q25–Q75) for non-normal distribution. AUC_∞_/DW: area under the plasma concentration–time curve from time zero to infinity, dose/weight corrected. AUC_72h_/DW: area under the plasma concentration–time curve from time zero to 72 h, dose/weight corrected. C_max_/DW: maximum plasma concentration, dose/weight corrected. t_1/2_: half-life. T_max_: time to reach maximum plasma concentration. NF: normal function. DF: decreased function. PF: poor function. RA: rapid acetylators. IA: intermediate acetylators. PA: poor acetylators. NM: normal metabolizer. IM: intermediate metabolizer. * *p* < 0.05 DF + PF compared to NF. *^1^ *p* < 0.05 compared to RA. *^2^ *p* < 0.05 compared to NM. *^3^ *p* < 0.05 compared to T/T. *^4^ *p* < 0.05 compared to C/C. *^5^ *p* < 0.05 compared to T/T. *^6^ *p* < 0.05 compared to C/C. *^7^ *p* < 0.05 compared to A/A. *^8^ *p* < 0.05 compared to A/A. *^9^ *p* < 0.05 G/A + A/A compared to G/G. *^10^ *p* < 0.05 G/A + G/G compared to A/A. *^11^ *p* < 0.05 compared to A/A. *^12^ *p* < 0.05 compared to G/G. ^@^ As it is not possible to obtain *p*-values from a single subject, the *p*-values shown are from the DF + PF analysis. ^@1^ As it is not possible to obtain *p*-values from a single subject, the *p*-values shown are from the IF + NF vs. DF + PF analysis. ^@2^ As it is not possible to obtain *p*-values from a single subject, the *p*-values shown are from the G/A + A/A analysis. ^#^ It is not possible to generate quartiles; therefore, the range of the data is shown for this result. Significant results are shown in bold. Underlined: multivariate *p*-value (*p_mv_*) < 0.05. ^$^ When compared in pairs is not significant. ^~^ T_max_ is constant.

**Table 4:** Rosuvastatin pharmacokinetic parameters without significance based on genotypes or phenotypes of genes involved in its transport or metabolism.

	N	AUC_∞_/DW (h*ng*kg/mL*mg)	AUC_72h_/DW (h*ng*kg/mL*mg)	C_max_/DW (ng*kg/mL*mg)	t_1/2_ (h)	T_max_ (h)
*ABCC2* rs2273697		*p =* 0.871	*p =* 0.879	*p =* 0.942	*p =* 0.686	*p =* 0.358
G/G	71	546.34 (232.35)	523.10 (228.94)	52.63 (23.38)	14.89 (12.59–19.50)	5.50 (5.50–5.50)
A/G	41	557.57 (225.76)	532.52 (218.03)	49.81 (21.73)	14.78 (12.94–19.89)	5.50 (4.50–5.50)
A/A	5	488.79 (94.50)	472.63 (89.41)	47.46 (10.17)	16.59 (15.12–17.23)	5.50 (3.50–5.50)
*ABCC2* rs3740066		*p =* 0.987	*p =* 0.980	*p =* 0.934	*p =* 0.068	*p =* 0.311
C/C	45	532.28 (161.66)	510.21 (157.18)	48.95 (17.96)	16.30 (13.73–22.35)	5.50 (4.75–5.50)
C/T	53	554.23 (244.48)	528.93 (240.61)	52.96 (24.89)	13.90 (12.56–16.48)	5.50 (3.75–5.50)
T/T	21	553.22 (285.92)	530.05 (277.91)	51.52 (23.92)	16.60 (12.07–21.77)	5.50 (5.50–5.50)
CYP2C19		*p =* 0.356	*p =* 0.333	*p =* 0.207	*p =* 0.764	*p =* 0.404
UM	4	677.43 (175.07)	645.20 (170.23)	62.77 (20.74)	15.60 (14.16–27.08)	5.50 (4.00–5.50)
RM	24	568.55 (279.70)	553.35 (277.71)	59.12 (30.15)	15.62 (13.07–18.08)	5.50 (3.50–5.50)
NM	53	512.33 (202.38)	487.90 (191.03)	46.84 (17.42)	14.72 (12.53–20.05)	5.50 (5.50–5.50)
IM	30	542.83 (206.91)	512.63 (207.35)	50.10 (21.34)	15.37 (12.46–22.07)	5.50 (4.00–5.50)
PM	2	783.44 (298.61)	764.74 (284.04)	75.37 (21.34)	12.52 (9.35–12.52) ^#^	5.00 (4.50–5.00) ^#^
CYP2C9		*p =* 0.361 ^@^	*p =* 0.349 ^@^	*p =* 0.715 ^@^	*p =* 0.973 ^@^	*p =* 0.126 ^@^
NM	81	523.17 (198.77)	500.30 (197.66)	49.73 (21.25)	15.82 (13.04–19.16)	5.50 (4.50–5.50)
IM	33	543.43 (197.37)	517.58 (185.38)	50.12 (19.59)	14.72 (10.08–22.10)	5.50 (2.70–5.50)
PM	1	1440.79	1356.70	92.08	20.24 ^#^	5.50 ^#^

Data are shown as mean (standard deviation) for normal distribution and median (Q25–Q75) for non-normal distribution. AUC_∞_/DW: area under the plasma concentration–time curve from time zero to infinity, dose/weight corrected. AUC_72h_/DW: area under the plasma concentration–time curve from time zero to 72 h, dose/weight corrected. C_max_/DW: maximum plasma concentration, dose/weight corrected. t_1/2_: half-life. T_max_: time to reach maximum plasma concentration. UM: ultrarapid metabolizer. RM: rapid metabolizer. NM: normal metabolizer. IM: intermediate metabolizer. PM: poor metabolizer. ^#^ It is not possible to generate quartiles; therefore, the range of the data is shown for this result. ^@^ As it is not possible to obtain *p*-values from a single subject, the *p*-values shown are from the IM + PM analysis.

## Data Availability

Data belong to the clinical trials’ sponsors and may be accessible upon reasonable request to the corresponding authors.

## Supplementary Materials

The following supporting information can be downloaded at https://www.mdpi.com/article/10.3390/ijms26010260/s1.
