# Supplementary material for: Effect of Genetic Variants on Rosuvastatin Pharmacokinetics in Healthy Volunteers: Involvement of ABCG2, SLCO1B1 and NAT2
Source: Int J Mol Sci. 2024 Dec 30;26(1):260. doi: 10.3390/ijms26010260 (PMC11720188; doi:10.3390/ijms26010260)
Supplement: Supplementary file 1 [file ijms-26-00260-s001.zip › ijms-3367514-supplementary.pdf]

EFFECT OF GENETIC VARIANTS ON ROSUVASTATIN PHARMACOKINETICS IN HEALTHY VOLUNTEERS: INVOLVEMENT OF *ABCG2*, *SLCO1B1* AND *NAT2*.

Eva González-Iglesias[1,2], Clara Méndez-Ponce[1], Dolores Ochoa[1,2], Manuel Román[1], Gina Mejía-Abril[1], Samuel Martín-Vilchez[1], Alejandro de Miguel[1], Antía Gómez-Fernández[1,2], Andrea Rodríguez-Lopez[1,2], Paula Soria-Chacartegui[1,2], Francisco Abad-Santos[1,2,3*], Jesús Novalbos[1*].

[1] Clinical Pharmacology Department, Hospital Universitario de La Princesa, Instituto de Investigación Sanitaria La Princesa (IIS-Princesa), 28006 Madrid, Spain.

[2] Pharmacology Department, Faculty of Medicine, Universidad Autónoma de Madrid (UAM), 28029 Madrid, Spain.

[3] Centro de Investigación Biomédica en Red de Enfermedades Hepáticas y Digestivas (CIBERehd), Instituto de Salud Carlos III, 28029 Madrid, Spain.

* Correspondence: francisco.abad@uam.es (F.A.-S.), jesus.novalbos@salud.madrid.org (J.N.)

Appendix A

Supplementary Table S1. Genes. SNPs and variants analysed in this study**.**

Gene	Alleles	SNP
ABCB1	N/A	rs1045642
	N/A	rs1128503
	N/A	rs2032582
ABCC2	N/A	rs2273697
	N/A	rs3740066
ABCC3	N/A	rs4793665
ABCG2	N/A	rs2231142
CES1	N/A	rs2244614
	N/A	rs8192936
COMT	N/A	rs13306278
	N/A	rs4680
	N/A	rs4818
	N/A	rs5993883
CYP1A2	N/A	rs2069514
	N/A	rs2069526
	N/A	rs2470890
	N/A	rs762551
CYP2A6	N/A	rs28399433
CYP2B6	*4, *5, *6, *7, *9, *18, *22, *34, *36	rs2279343
		rs28399499
		rs3211371
		rs34223104
		rs3745274
CYP2C18	N/A	rs11188059
	N/A	rs2860840
CYP2C19	*2, *3, *4, *5, *6, *7, *8, *9, *17, *35	rs12248560
		rs12769205
		rs17884712
		rs28399504
		rs41291556
		rs4244285
		rs4986893
		rs56337013
		rs72552267
		rs72558186

CYP2C8	*2, *3, *4	rs10509681
		rs1058930
		rs11572080
		rs11572103
CYP2C9	*2, *3, *5, *6, *8, *11, *45	rs1057910
		rs1799853
		rs199523631
		rs28371685
		rs28371686
		rs7900194
		rs9332131
CYP2D6	*2, *3, *4, *5, *6, *7, *8, *9, *10, *12, *14, *15, *17, *19, *29, *31, *41, *42, *49, *56, *59, *64, *65, *69, *107, *109, *114, *119	rs1065852
		rs1135822
		rs1135840
		rs16947
		rs267608319
		rs28371706
		rs28371725
		rs35742686
		rs3892097
		rs5030655
		rs5030656
		rs5030862
		rs5030865
		rs5030867
		rs59421388
		rs61736512
		rs72549346
		rs72549347
		rs72549353
		rs774671100
		rs79292917
CYP3A4	*2, *3, *4, *5, *6, *18, *20, *22, *37	rs2242480
		rs2740574
		rs28371759
		rs35599367
		rs4646438
		rs4986910

		rs55785340
		rs55901263
		rs55951658
		rs67666821
CYP3A43	*2	rs61469810
CYP3A5	*3, *6, *7	rs776746
		rs10264272
		rs41303343
CYP4F2	*2, *3, *4, *5, *6, *7	rs2108622
		rs3093105
		rs3093153
		rs3093200
G6PD	N/A	rs1050828
	N/A	rs1050829
	N/A	rs137852318
MTHFR	N/A	rs1801131
	N/A	rs1801133
NAT2	*5, *6, *7	rs1801280
		rs1799930
		rs1799931
NUDT15	*3	rs116855232
SLC19A1	N/A	rs1051266
SLC22A1	N/A	rs628031
	N/A	rs72552763
	N/A	rs12208357
	N/A	rs34059508
SLC22A2	N/A	rs316019
SLC28A3	N/A	rs7853758
SLC6A2	N/A	rs3785143
	N/A	rs12708954
SLCO1B1	*4, *5, *9, *14, *15, *19, *20, *23, *31, *37, *40	rs11045819
		rs2306283
		rs34671512
		rs373327528
		rs4149056
		rs59502379
TPMT	*2, *3A, *3B, *3C, *4, *11	rs1142345

		rs1800460
		rs1800462
		rs1800584
		rs72552738
UGT1A	N/A	rs10929302
UGT1A1	*80	rs887829
UGT1A34	N/A	rs2008584
UGT1A4	N/A	rs2011425
UGT1A6	N/A	rs7592281
	N/A	rs10445704
UGT1A8	N/A	rs1042597
UGT2B10	N/A	rs61750900
UGT2B15	N/A	rs1902023
UGT2B7	N/A	rs7668258

Supplementary Table S2. Rosuvastatin pharmacokinetic data of study population by genotype or phenotype for all screened genes.

	N	AUC_{∞}/DW (h*ng*kg/mL*mg)	AUC_{72h}/DW (h*ng*kg/mL*mg)	C_{max}/DW (ng*kg/mL*mg)	$T_{1/2}$ (h)	T_{max} (h)
ABCB1 rs1045642		*p*=0.851	*p*=0.934	*p*=0.936	*p*=0.586	*p*=0.291
T/T	28	551.27 (302.91)	535.10 (302.41)	52.93 (31.69)	14.42 (12.69-19.17)	5.50 (5.50-5.50)
C/T	62	543.60 (210.58)	519.01 (200.42)	50.46 (19.25)	15.88 (12.76-19.99)	5.50 (3.50-5.50)
C/C	29	545.02 (160.64)	515.95 (161.05)	51.05 (17.32)	15.23 (13.13-19.10)	5.50 (5.50-5.50)
ABCB1 rs1128503		*P*=0.215	*P*=0.296	*P*= 0.261	*P*= 0.265	*P*= 0.132
T/T	16	589.05 (346.10)	573.06 (346.30)	57.24 (38.04)	13.73 (11.88-16.73)	5.50 (5.50-5.50)
T/C	63	508.48 (177.66)	486.70 (170.39)	47.74 (18.71)	15.90 (13.08-18.81)	5.50 (3.50-5.50)
C/C	40	587.12 (224.55)	557.32 (219.74)	54.21 (18.34)	15.31 (13.11-21.85)	5.50 (5.50-5.50)
ABCB1 rs2032582		*p*=0.305	*p*=0.302	*p*= 0.383	*p*= 0.628	*p*=0.602
T/T	14	586.68 (370.11)	570.85 (370.59)	57.13 (40.33)	14,42 (12.52-16.74)	5,5 (5.50-5.50)
T/G	57	544.00 (191.50)	521.31 (184.64)	51.00 (19.82)	16.00 (12.98-18.82)	5,5 (3.50-5.50)
T/A	3	330.06 (122.65)	305.97 (101.95)	31.15 (14.22)	14,37 (12.42-31.27)[#]	5,5 (3.00-5.50)[#]
G/A	38	560.03 (218.89)	531.34 (211.60)	51.48 (17.58)	14,70 (12.56-20.76)	5,5 (5.50-5.50)
G/G	6	490.62 (125.20)	470.28 (131.31)	46.74 (16.83)	18,23 (13.59-23.13)	5,5 (3.79-5.63)
ABCC3 rs4793665		*p*=0.111	*p*=0.118	*p*=0.080	*p*=0.739	*p*=0.147
C/C	14	632.85 (172.50)	612.46 (171.69)	62.18 (19.65)	17.26 (12.60-19.86)	5.00 (2.75-5.50)
C/T	55	522.57 (230.09)	503.49 (224.68)	49.37 (23.86)	14.78 (13.08-17.32)	5.50 (5.00-5.50)
T/T	50	546.86 (226.99)	517.15 (221.70)	50.12 (20.47)	14.81 (12.63-20.76)	5.50 (5.50-5.50)
CES1 rs2244614		*p*=0.784	*p*=0.864	*p*=0.553	*p*=0.789	*p*=0.338
C/C	3	454.18 (44.61)	430.97 (65.88)	37.55 (14.29)	15.66 (13.65-15.66)[#]	5.50 (5.50-5.50)[#]
C/A	36	557.20 (216.14)	522.89 (210.15)	50.18 (21.19)	15.06 (12.29-15.06)	5.50 (5.13-5.50)
A/A	80	544.03 (231.59)	525.09 (227.04)	52.16 (22.90)	15.01 (12.92-19.34)	5.50 (4.13-5.50)
COMT rs4680		*p*=0.410	*p*=0.272	*p*=0.147	*p*=0.121	*p*=0.906
G/G	37	540.84 (253.69)	519.64 (244.02)	50.34 (21.22)	15.82 (13.27-20.57)	5.50 (3.75-5.50)
G/A	60	561.63 (192.89)	540.04 (188.19)	53.63 (20.32)	14.42 (12.43-16.98)	5.50 (4.63-5.50)
A/A	22	510.68 (253.63)	477.05 (254.00)	45.95 (28.18)	17.20 (13.14-20.06)	5.50 (5.50-5.50)
COMT rs4818		*p*=0.708	*p*=0.617	*p*=0.956	*p*=0.670	*p*=0.206
C/C	53	526.07 (210.60)	501.09 (210.81)	51.15 (23.78)	15.96 (13.09-20.65)	5.50 (5.50-5.50)
C/G	49	562.76 (219.05)	539.25 (212.67)	51.94 (21.56)	14.89 (12.78-16.99)	5.50 (4.00-5.50)
G/G	13	591.54 (311.93)	569.24 (295.29)	50.99 (20.15)	15.42 (11.79-18.31)	5.50 (5.50-5.50)
COMT rs5993883		*p*=0.467	*p*=0.608	*p*=0.377	*p*=0.517	*p*=0.839
T/T	33	546.82 (163.50)	517.14 (169.30)	54.54 (21.09)	16.30 (13.09-22.42)	5.50 (5.50-5.50)
T/G	51	526.51 (227.51)	508.95 (223.64)	49.35 (22.99)	15.23 (13.08-18.15)	5.50 (4.00-5.50)
G/G	35	572.77 (266.69)	545.77 (254.66)	50.71 (22.37)	14.39 (11.98-19.91)	5.50 (4.00-5.50)
CYP1A2 rs2069514		*p*=0.071	*p*=0.116	*p*=0.661	*p*=0.527	***p*=0.034[&]**
G/G	84	572.28 (225.18)	545.41 (220.55)	52.48 (22.49)	15.23 (12.51-19.77)	5.50 (4.00-5.50)
G/A	24	473.82 (170.73)	455.19 (165.41)	47.23 (17.66)	15.86 (13.61-19.51)	5.50 (5.50-5.50)
A/A	9	503.05 (306.22)	493.83 (307.03)	52.94 (31.36)	14.10 (12.08-16.81)	5.50 (5.50-5.75)
CYP1A2 rs2069526		*p*=0.179	*p*=0.476	*p*=0.910	*p*=0.312	*p*=0.914
T/T	112	538.11 (212.11)	517.19 (208.37)	51.23 (22.32)	15.06 (13.03-18.66)	5.00 (5.50-5.50)
G/T	6	700.80 (388.82)	622.85 (391.33)	50.10 (24.28)	20.05 (12.32-43.82)	5.00 (5.50-5.50)

CYP1A2 rs2470890		p=0.259	p=0.156	p=0.272	p=0.085	p=0.078
T/T	23	545.34 (151.52)	521.71 (148.29)	50.44 (16.38)	16.34 (14.18-20.89)	5.50 (3.50-5.50)
T/C	51	574.61 (250.96)	555.85 (244.62)	53.79 (22.29)	13.89 (12.42-18.15)	5.00 (5.50-5.50)
C/C	43	512.44 (218.52)	482.43 (212.81)	48.69 (24.53)	16.24 (13.30-22.37)	5.00 (5.50-5.50)
CYP1A2 rs762551		p=0.686	p=0.616	p=0.446	p=0.992	p=0.835
C/C	17	511.66 (216.73)	478.98 (198.61)	47.05 (25.46)	14.37 (12.29-22.84)	5.50 (3.78-5.50)
C/A	48	559.39 (229.81)	533.86 (231.34)	52.38 (24.24)	15.31 (12.91-19.51)	5.50 (4.13-5.50)
A/A	54	544.35 (223.33)	525.11 (215.74)	51.44 (19.37)	15.33 (13.07-18.35)	5.50 (5.50-5.50)
CYP2A6 rs28399433		p=0.125	p=0.162	p=0.356	p=0.915	p=0.696
A/A	102	556.45 (228.42)	531.53 (223.54)	51.77 (22.62)	14.84 (12.87-19.59)	5.50 (4.88-5.50)
A/C	16	481.22 (192.27)	465.48 (189.92)	47.20 (20.42)	15.89 (12.46-17.94)	5.50 (4.63-5.50)
CYP2B6		p=0.194	p=0.166	p=0.318	p=0.257	**p=0.016[&]**
RM	4	372.61 (134.81)	360.45 (132.26)	36.90 (9.06)	16.52 (14.87-17.16)	5.75 (5.50-6.00)
NM	55	570.19 (245.38)	545.13 (236.84)	54.04 (23.67)	15.85 (12.87-19.91)	5.50 (3.50-5.50)
IM	44	510.40 (163.94)	482.85 (160.65)	47.17 (17.58)	15.14 (12.92-21.88)	5.50 (5.50-5.50)
PM	14	613.63 (300.09)	603.03 (299.52)	58.10 (29.85)	14.16 (11.49-15.38)	5.50 (5.50-5.50)
CYP2C18 rs11188059		p=0.066	p=0.091	p=0.075	p=0.355	p=0.258
G/G	87	568.58 (235.77)	541.87 (230.34)	53.18 (23.27)	15.42 (13.07-20.24)	5.50 (4.50-5.50)
A/G	28	470.26 (181.22)	454.52 (181.43)	44.48 (19.11)	14.57 (11.90-18.78)	5.50 (5.50-5.50)
A/A	4	577.57 (100.22)	563.64 (102.63)	54.85 (6.41)	14.17 (9.18-16.14)	5.50 (4.38-5.50)
CYP2C18 rs2860840		p=0.453	p=0.439	p=0.323	p=0.546	p=0.098
C/C	45	592.52 (287.09)	568.87 (279.55)	56.11 (27.82)	14.67 (12.76-18.18)	5.50 (3.09-5.50)
C/T	58	523.64 (180.47)	498.42 (177.44)	48.80 (18.12)	15.54 (13.13-21.25)	5.50 (5.50-5.50)
T/T	14	484.79 (135.20)	466.90 (135.41)	44.74 (15.02)	14.73 (10.64-18.38)	5.50 (5.50-5.50)
CYP2C8		p=0.598[@]	p=0.597[@]	p=0.531[@]	p=0.322[@]	p=0.328[@]
*1/*1	85	552.16 (232.66)	527.33 (226.48)	52.19 (23.03)	14.45 (12.62-17.97)	5.50 (5.50-5.50)
*1/*2	1	590.23	563.82	55.59	20.41[#]	4.00[#]
*2*3	1	278.03	273.61	29.96	13.14[#]	5.50[#]
*1/*3	20	551.56 (201.18)	530.05 (199.03)	51.33 (20.70)	15,67 (12.80-23.08)	5,50 (3,75-5.50)
*1/*4	7	532.58 (158.91)	507.31 (164.64)	44.48 (13.80)	18,19 (15,90-28.27)	4,50 (1.50-5.50)
*3/*3	3	370.07 (72.90)	353.90 (75.68)	32.16 (7.18)	15,42 (10.70-24.99)[#]	5,50 (5.50-5.50)
*4/*4	1	290.30	276.45	37.94	15.85[#]	2.50[#]
CYP2D6		p=0.092	p=0.204	p=0.292	p=0.599	p=0.239
UM	8	435,52 (143,83)	381,46 (117,39)	41,48 (18,11)	19,49 (13,41-24,41)	5,50 (5,50-5,50)
NM	64	550,14 (205,77)	527,77 (201,00)	52,04 (21,64)	15,06 (13,17-19,34)	5,50 (5,50-5,50)
IM	35	550,20 (241,73)	528,44 (232,28)	50,66 (21,68)	14,37 (12,64-18,17)	5,50 (4,50-5,50)
PM	5	743,99 (407,36)	724,64 (410,32)	68,87 (40,78)	14,72 (11,89-22,32)	5,50 (3,50-5,50)
CYP3A4 rs2242480		p=0.110	p=0.137	p=0.330	p=0.876	p=0.067
G/G	68	567.56 (238.04)	546.90 (232.76)	52.08 (22.97)	15.40 (13.03-18.66)	5.50 (3.63-5.50)
G/A	41	494.87 (173.65)	470.53 (162.47)	47.84 (17.94)	14.78 (12.39-21.12)	5.50 (5.50-5.50)
A/A	9	642.44 (275.95)	596.58 (296.61)	62.08 (31.55)	13.89 (12.73-19.12)	5.50 (5.50-5.75)
CYP3A4 rs2740574		p=0.299	p=0.134	p=0.219	p=0.800	p=0.287
G/A	21	518.29 (257.49)	476.54 (246.17)	46.11 (24.27)	16.00 (11.80-22.40)	5.50 (5.50-5.50)
A/A	97	548.97 (216.35)	529.16 (212.15)	52.04 (21.71)	14.89 (13.04-18.18)	5.50 (4.25-5.50)

CYP3A4		p=0.398	p=0.429	p=0.360	p=0.573	p=0.431
NM	107	548.90 (228.12)	524.72 (223.67)	51.68 (22.71)	15.66 (13.08-19.51)	5.50 (4.50-5.50)
IM	5	459.46 (159.80)	442.65 (154.22)	42.69 (17.23)	14.37 (12.41-20.88)	5.50 ~
CYP3A5		p=0.416[@1]	p=0.492[@1]	p=0.597[@1]	p=0.355[@1]	p=0.511[@1]
NM	1	344.49	316.00	28.94	22.33[#]	4.00[#]
IM	21	518.77 (211.90)	501.39 (209.16)	49.83 (20.66)	14,10 (12.65-17.28)	5.50 (5.50-5.50)
PM	97	553.66 (226.99)	528.65 (221.77)	51.71 (22.62)	15,66 (12.94-19.69)	5.50 (4.75-5.50)
CYP4F2 rs3093153		p=0.417	p=0.421	p=0.230	p=0.073	p=0.485
G/G	107	542.03 (228.33)	518.14 (222.56)	50.41 (22.12)	15.42 (13.10-19.51)	5.50 (5.00-5.50)
T/G	12	578.91 (182.17)	556.94 (187.25)	58.11 (22.84)	12.53 (11.20-18.22)	5.50 (4.63-5.50)
CYP4F2 rs3093200		p=0.302	p=0.387	p=0.370	p=0.186	p=0.365
C/C	100	556.43 (235.16)	530.96 (230.17)	52.27 (23.48)	15.31 (13.15-19.77)	5.50 (5.13-5.50)
C/A	18	473.19 (125.72)	458.44 (124.16)	43.99 (11.25)	13.62 (11.50-17.89)	5.50 (4.13-5.50)
G6PD rs137852318		p=0.905	p=0.858	p=0.891	p=0.909	p=0.696
C/C	116	541.35 (220.24)	517.58 (215.31)	50.90 (22.06)	15.06 (12.87-19.51)	5.50 (4.63-5.50)
C/G	2	576.22 (350.02)	559.22 (345.57)	48.27 (26.32)	15.37 (14.37-16.37)[#]	5.50 (5.50-5.50)
MTHFR rs1801131		p=0.806	p=0.858	p=0.791	p=0.266	p=0.167
A/A	69	542.29 (195.123)	515.58 (192.19)	52.37 (22.33)	15.38 (13.33-19.16)	5.50 (5.50-5.50)
A/C	43	547.93 (272.20)	529.61 (265.55)	49.39 (23.17)	14.37 (12.63-19.51)	5.50 (4.00-5.50)
C/C	5	589.61 (209.79)	557.05 (195.62)	48.99 (13.09)	18.17 (13.09-29.33)	5.50 (3.50-5.50)
MTHFR rs1801133		p=0.848	p=0.856	p=0.790	p=0.509	p=0.414
C/C	33	533.18 (222.80)	507.50 (211.40)	47.20 (16.25)	15.66 (13.52-21.29)	5.50 (4.25-5.50)
C/T	61	540.73 (231.92)	519.62 (232.06)	51.69 (23.78)	14.78 (12.61-17.53)	5.50 (5.25-5.50)
T/T	24	561.99 (205.11)	534.54 (194.69)	53.51 (23.89)	14.59 (13.08-20.22)	5.50 (5.50-5.50)
NUDT15		p=0.387	p=0.998	p=0.814	p=0.083	p=0.558
NM	115	544.90 (226.08)	523.63 (220.71)	51.25 (22.37)	14.89 (12.87-19.50)	5.50 (5.00-5.50)
IM	3	627.83 (135.30)	507.14 (199.24)	53.05 (22.16)	18.15 (16.82-108.02)[#]	5.50 (1.50-5.50)[#]
SLC19A1 rs1051266		p=0.366	p=0.348	p=0.369	p=0.760	p=0.416
A/A	24	508.83 (190.08)	488.01 (186.70)	48.75 (21.01)	16.19 (13.00-21.48)	5.50 (4.63-5.50)
G/A	61	538.63 (224.12)	512.69 (218.89)	49.88 (22.50)	15.23 (12.53-18.85)	5.50 (4.00-5.50)
G/G	33	586.10 (248.23)	563.94 (242.69)	55.18 (22.97)	14.68 (13.19-19.87)	5.50 (5.50-5.50)
SLC22A1 rs12208357		p=0.528	p=0.568	p=0.692	p=0.907	p=0.812
C/C	112	550.22 (228.99)	526.02 (224.07)	51.55 (22.60)	15.23 (12.91-19.33)	5.50 (5.13-5.50)
C/T	6	463.38 (92.38)	446.73 (92.84)	45.70 (16.02)	15.28 (10.74-25.26)	5.50 (2.75-5.63)
SLC22A1 rs34059508		p=0.664	p=0.588	p=0.955	p=0.151	p=0.215
G/G	117	544.69 (223.77)	520.70 (218.71)	51.14 (22.14)	15.23 (13.04-19.51)	5.50 (4.75-5.50)
A/G	2	607.53 (299.75)	600.76 (301.04)	54.20 (36.61)	12.34 (11.80- 12.34)[#]	5.75 (5.50-5.75)[#]
SLC22A1 rs628031		p=0.921	p=0.885	p=0.626	p=0.301	p=0.418
A/A	16	553.18 (190.03)	515.35 (172.20)	46.04 (18.85)	16.50 (12.69-26.81)	5.50 (5.50-5.50)
G/A	45	559.26 (267.11)	540.92 (259.12)	52.59 (23.81)	14.67 (12.45-18.84)	5.50 (3.50-5.50)
G/G	57	531.94 (198.27)	507.73 (199.06)	51.03 (21.81)	15.66 (13.12-18.82)	5.50 (5.25-5.50)
SLC22A1 rs72552763		p=0.815	p=0.809	p=0.959	p=0.190	p=0.142
GAT/GAT	79	555.46 (240.21)	531.15 (234.94)	51.87 (23.49)	14.68 (12.42-18.82)	5.50 (4.50-5.50)
-/GAT	32	526.35 (202.66)	503.29 (197.55)	50.31 (21.27)	15.22 (13.17-21.71)	5.50 (5.50-5.50)
-/-	8	527.42 (125.39)	507.21 (129.47)	48.01 (12.14)	16.59 (16.32-17.85)	5.25 (2.25-5.50)

SLC22A2 rs316019		p=0.298	p=0.410	p=0.295	p=0.567	p=0.827
T/T	2	575.90 (228.36)	566.96 (225.15)	65.80 (23.19)	13.18 (12.47-13.18)#	5.50 ~
T/G	16	613.57 (246.89)	577.83 (254.66)	56.30 (25.36)	14.82 (12.47-21.36)	5.50 (5.50-5.50)
G/G	101	534.41 (220.26)	512.32 (213.67)	50.09 (21.70)	15.23 (13.09-19.51)	5.50 (4.50-5.50)
SLC28A3 rs7853758		p=0.413	p=0.738	p=0.837	p=0.794	p=0.294
C/C	74	536.31 (189.53)	513.00 (183.25)	49.94 (19.85)	15.74 (12.87-20.00)	5.50 (4.50-5.50)
C/T	36	541.68 (254.98)	523.70 (246.64)	52.09 (22.39)	14.68 (12.76-17.63)	5.50 (5.50-5.50)
T/T	8	669.96 (349.60)	615.42 (373.59)	60.27 (39.57)	14.13 (13.03-27.86)	5.25 (3.38-5.50)
SLC6A2 rs12708954		p=0.930	p=0.930	p=0.851	p=0.610	p=0.777
C/C	83	542.88 (198.21)	520.34 (195.94)	51.14 (21.02)	14.68 (13.08-17.79)	5.50 (5.00-5.50)
A/C	29	564.83 (297.41)	537.79 (288.87)	52.05 (27.66)	15.96 (12.39-22.35)	5.50 (5.00-5.50)
A/A	6	549.83 (127.29)	523.63 (104.54)	51.27 (4.49)	17.90 (13.39-23.83)	5.50 (3.75-5.50)
SLC6A2 rs3785143		p=0.115	p=0.129	p=0.085	p=0.276	p=0.709
C/C	102	557.64 (227.80)	532.63 (223.28)	52.39 (22.51)	15.40 (13.06-19.61)	5.50 (4.50-5.50)
C/T	15	450.04 (175.23)	432.98 (167.93)	40.99 (15.84)	14.25 (12.42-16.82)	5.50 (5.50-5.50)
T/T	2	657.14 (242.07)	650.40 (242.21)	66.41 (37.02)	12.15 (11.00-12.45)#	5.50 (5.00-5.50)#
UGT1A rs10929302		p=0.269	p=0.366	p=0.803	p=0.209	p=0.051
G/G	61	564.43 (220.37)	538.70 (219.12)	52.82 (23.92)	15.82 (13.08-19.71)	5.50 (4.00-5.50)
G/A	49	544.25 (239.94)	520.72 (231.61)	50.19 (21.22)	14.37 (12.53-20.05)	5.50 (5.50-5.50)
A/A	9	427.29 (100.80)	416.44 (99.41)	45.63 (15.02)	13.89 (11.28-16.28)	5.50 (3.03-5.50)
UGT1A4 rs2011425		p=0.346	p=0.617	p=0.237	p=0.460	p=0.317
T/T	103	537.96 (226.39)	517.69 (220.65)	50.25 (21.83)	14.89 (12.63-18.19)	5.50 (5.50-5.50)
T/G	15	586.88 (209.16)	543.63 (217.38)	57.33 (25.25)	15.85 (13.10-25.33)	5.50 (4.00-5.50)
UGT1A6 rs7592281		p=0.838	p=0.740	p=0.764	p=0.215	p=0.564
G/G	117	545.41 (224.33)	521.42 (219.41)	51.09 (22.13)	15.23 (12.94-19.51)	5.50 (5.50-5.50)
G/T	2	565.66 (260.20)	559.16 (254.78)	57.33 (36.39)	12.40 (11.00- 12.40)#	4.50 (3.50-4.50)#
UGT1A8 rs1042597		p=0.638	p=0.649	p=0.835	p=0.299	p=0.762
C/C	63	563.89 (247.99)	540.56 (242.67)	52.33 (24.45)	14.68 (12.63-18.82)	5.50 (5.00-5.50)
C/G	47	521.48 (195.67)	501.07 (189.42)	49.87 (18.82)	15.82 (13.65-20.41)	5.50 (5.50-5.50)
G/G	9	545.48 (188.21)	502.06 (195.21)	50.10 (24.25)	13.76 (12.00-14.75)	5.50 (3.84-5.50)
*UGT2B10*_rs61750900		p=0.601	p=0.689	p=0.630	p=0.421	p=0.094
G/G	105	545.42 (211.43)	521.03 (208.31)	51.43 (22.46)	15.23 (12.94-19.69)	5.50 (4.50-5.50)
G/T	14	548.21 (310.93)	529.72 (295.92)	49.40 (20.97)	14.49 (12.30-17.65)	5.50 (5.50-5.63)
UGT2B7 rs7668258		p=0.080	p=0.096	p=0.341	p=0.821	p=0.495
T/T	21	583.04 (201.37)	563.96 (203.63)	54.67 (25.13)	13.89 (12.40-19.54)	5.50 (4.25-5.50)
T/G	64	511.75 (222.13)	488.78 (209.47)	48.36 (19.20)	15.46 (12.70-19.77)	5.50 (5.50-5.50)
G/G	34	586.71 (235.25)	558.79 (240.25)	54.37 (25.38)	15.31 (13.06-17.15)	5.50 (4.75-5.50)

Data are shown as mean (standard deviation) for normal distributions and median (Q25-Q75) for non-normal distributions. AUC_{∞}/DW: Area under the plasma concentration-time curve from time zero to infinity, dose/weight corrected. AUC_{72h}/DW: Area under the plasma concentration-time curve from time zero to 72 hours, dose/weight corrected. C_{max}/DW: Maximum plasma concentration, dose/weight corrected. $t_{1/2}$: Half-life. T_{max}: Time to reach maximum plasma concentration. RM: rapid metabolizer. UM: ultrarrapid metabolizer. NM: normal metabolizer. IM: intermediate metabolizer. PM: poor metabolizer. # It is not possible to generate quartiles, therefore, the range of the data is shown for this result. ~ T_{max} is constant. & not significant when comparing pairs. @ As it is not possible

*to obtain p-values from a single subject, the p-values shown are from the *1/*2+*2/*3 and *1/*4+*4/*4 analysis.*

@[1] As it is not possible to obtain p-values from a single subject, the p-values shown are from the NM+IM analysis.

@[2] As it is not possible to obtain p-values from a single subject, the p-values shown are from the A/G+G/G analysis.

@[3] As it is not possible to obtain p-values from a single subject, the p-values shown are from the C/T+T/T analysis.
